# A Parallel Software Pipeline for DMET Microarray Genotyping Data Analysis

**DOI:** 10.3390/ht7020017

**Published:** 2018-06-14

**Authors:** Giuseppe Agapito, Pietro Hiram Guzzi, Mario Cannataro

**Affiliations:** Data Analytics Research Center, Department of Medical and Surgical Sciences, University “Magna Græcia” of Catanzaro, Viale Europa, 88100 Catanzaro, Italy; agapito@unicz.it (G.A.); hguzzi@unicz.it (P.H.G.)

**Keywords:** single nucleotide polymorphisms, multiple analysis pipeline, pharmacogenomics, overall survival curves, data mining, statistical analysis

## Abstract

Personalized medicine is an aspect of the P4 medicine (predictive, preventive, personalized and participatory) based precisely on the customization of all medical characters of each subject. In personalized medicine, the development of medical treatments and drugs is tailored to the individual characteristics and needs of each subject, according to the study of diseases at different scales from genotype to phenotype scale. To make concrete the goal of personalized medicine, it is necessary to employ high-throughput methodologies such as Next Generation Sequencing (NGS), Genome-Wide Association Studies (GWAS), Mass Spectrometry or Microarrays, that are able to investigate a single disease from a broader perspective. A side effect of high-throughput methodologies is the massive amount of data produced for each single experiment, that poses several challenges (e.g., high execution time and required memory) to bioinformatic software. Thus a main requirement of modern bioinformatic softwares, is the use of good software engineering methods and efficient programming techniques, able to face those challenges, that include the use of parallel programming and efficient and compact data structures. This paper presents the design and the experimentation of a comprehensive software pipeline, named microPipe, for the preprocessing, annotation and analysis of microarray-based Single Nucleotide Polymorphism (SNP) genotyping data. A use case in pharmacogenomics is presented. The main advantages of using microPipe are: the reduction of errors that may happen when trying to make data compatible among different tools; the possibility to analyze in parallel huge datasets; the easy annotation and integration of data. microPipe is available under Creative Commons license, and is freely downloadable for academic and not-for-profit institutions.

## 1. Introduction

The continuous improvements in experimental technologies allow spreading the use of genotyping analysis in several biological, medical and clinical areas [1,2]. The investigation of complex diseases such as cancer, Alzheimer,’s and leukemia, requires to investigate multiple actors (i.e., genes, proteins and small molecules) at the same time. Thus, it is necessary to use high-throughput experimental platforms such as Next Generation Sequencing (NGS), Genome-Wide Association Studies (GWAS), Mass Spectrometry and Microarrays. GWAS offers a large-scale genotyping of Single Nucleotide Polymorphisms (SNPs) in thousands of DNA samples and it is one of the most powerful methods for recognizing genes related to complex diseases as reported in [3]. SNP microarrays have been used to identify the genetic polymorphisms involved in drug transportation and metabolization that play a crucial function in the efficacy and toxicity of treatment based on erlotinib [4]. In this study [5,6], the effectiveness of using Drug Metabolism Enzymes and Transporters (DMET) microarray in a clinical trial has been proven. DMET microarrays are employed to investigate SNPs correlated to Absorption, Distribution, Metabolism, and Excretion (ADME) genes contributing to the different drug sensitivity, resistance, and toxicity of patients [7].

A side effect of high-throughput methodologies is the massive amount of data produced per single experiment, posing several challenges regarding execution time and required memory. Also, the format used to code the experimental data is not suitable to be directly used as input with the most used data mining and statistical software available, e.g., Weka (https://www.cs.waikato.ac.nz/ml/weka/downloading.html), SPSS (https://www.ibm.com/analytics/data-science/predictive-analytics/spss-statistical-software), R (https://www.r-project.org) and many others. Thus, a lot of efforts to make datasets compatible with the chosen tool are needed in order to make it possible to extract actionable knowledge from these data.

Nowadays, no comprehensive framework with which to perform both statistical and/or data mining analysis is available. For example, to conduct a statistical analysis with SPSS or R by using as input a DMET SNP dataset, researchers have to convert the literals SNPs contained into the dataset in numbers to be compatible with R and SPSS to start the analysis. This conversion is mandatory and has to be done manually, making it a tedious and error-prone task. Moreover, to analyze the same dataset from a data mining perspective, it is necessary to further manipulate the original dataset. For instance, to extract association rules from a DMET SNP dataset by using Weka, researchers have to convert the dataset in the Attribute-Relation File Format (ARFF) format, or alternatively in Comma Separated Values (CSV).

Modern bioinformatic softwares should be developed through the use of efficient programming paradigms able to face those challenges, which include the use of parallel computing and efficient data structures. Providing tools simply to use even for those scientists with basic or none programming skills, or even with limited time, that look for straightforward solutions with low time investment.

In previous works, we developed some tools to perform statistical and data mining analysis of DMET datasets. DMET-Analyzer [8] is a software tool able to perform statistical analysis of DMET SNP datasets to discriminate single SNPs involved in adverse drug reaction. DMET-Miner [9] is a software to extract association rules able to link together multiple SNPs involved in adverse drug reactions. Finally, OS-Analyzer (OSA) is a software tool for the analysis of DMET datasets annotated with clinical data such as overall survival (OS), progression-free survival (PFS), to graphically discriminate which SNP is related to a good or bad overall survival.

To perform data mining, temporal and statistical analysis, users have to manually compose a pipeline with the three softwares. To perform data mining, survival and statistical analysis of the same DMET SNP dataset, users have to perform multiple executions a long and laborious process. To speed up and simplify the analysis of DMET SNP datasets, we present *microPipe* a novel tool to perform on the same input dataset statistical, survival and data mining analysis, integrating the functions of DMET-Analyzer, DMET-Miner, and OS-Analyzer. The main advantage of microPipe is the possibility to perform data mining, statistical and temporal analysis on the same dataset in a single execution. microPipe makes the multiple analysis of DMET datasets faster since, the only operation required from the users is to load the input dataset.

The remainder of the manuscript is structured ad follows: Section 2 describes previous and related work on the DMET analysis tools. Section 3 presents microPipe, Section 4 reports the main steps of microPipe. Finally, Section 5 concludes the manuscript.

## 2. Related Work

Recently, microarrays have become one of the most suitable methodologies in genomic research, due to the high magnitude of simultaneously analyzed genes per single experiment. Microarrays are involved in many life science areas, spurring for the development of suitable data analysis models and tools. On the other hand, the necessity to provide researchers with software tools that are simple to use, arises as well.

In this Section, we summarize the software tools listed on the OMICtools (https://omictools.com) website and evaluated as compatible with DMET data, and the software tools able to deal with DMET SNP microarray datasets developed by our research group. We assessed tools that can extract knowledge from DMET SNP datasets and tools that can do only modelling and prediction of the structure of DMET enzyme.

- DMET-Analyzer [8] is a tool for the automatic association analysis of the difference between the genomics characteristics and the clinical status of patients, e.g., the different response to drugs. DMET-Analyzer can investigate the association between the presence or absence of SNP related to the groups of patients (e.g., RESPONDER or NOT-RESPONDER) by using the well known Fisher’s test. To improve the statistical significance of results DMET-Analyzer implements the Bonferroni and False Discovery Rate (FDR) statistical correctors. Finally, DMET-Analyzer can retrieve information provided by Affymetrix libraries and with links to the dbSNP database and the PharmGKB pharmacogenomics database to annotates the computed relevant SNPs.
- DMET-Miner [9] is a tool developed to automatically and easily mine association rules from DMET datasets that can highlight the presence–absence of multiple SNPs related to the clinical condition of a subject. For example, the combination of SNPs responsible for the different response to drugs by an individual.
- OS-Analyzer [10] is a software tool to analyze DMET datasets annotated with clinical data such as OS and PFS. The primary function of OSA is the automatic computation and visualization of OS and PFS curves due to the presence of specific ADME genes, from a whole DMET dataset. Results are conveyed to the user ranked by statistical relevance obtained computed the area under the curves by using log-rank test.
- coreSNP [11] is the parallel version of DMET-Analyzer. coreSNP is a tool for the parallel association analysis of the SNPs variation of the patient and the clinical conditions of patients (e.g., the different response to treatments), computed by implementing the well known Fisher’s test. coreSNP to improve the statistical relevance of results, the Bonferroni and False Discovery Rate correctors are available. Finally, results are visualized as a heat map to give fast visual feedback, allowing users to interpret the results instantly.
- PARES (Parallel Association Rules Extractor from SNPs) [12] is a software tool for the parallel extraction of association rules, through a multi-thread version of an customised version of the Frequent Pattern Growth (FP-Growth) algorithm. PARES comes with a simple and intuitive graphic user interface, allowing to the user with basic or none computer science skills to mine relations between SNPs and the clinical condition of patients hidden in DMET datasets.
- Cloud4SNP [13] is a Cloud-based tool for the statistical analysis of DMET dataset. It is a Cloud-based version of DMET-Analyzer that has been realized on the Cloud by using the Data Mining Cloud Framework [14], a software environment to design and execute analysis workflows on the Cloud [15]. Cloud4SNP through the Fisher’s Test allows users to test the statistical relevance of the presence of multiple SNPs per time in a class and the clinical condition of a sample.
- ADMET Predictor is a software tool to predict the 2D structure of the molecule to create high-quality models with which to produce compounds. Molecules’ structure modeling is done through a graphical user interface. ADMET Predictor is available for Unix/Linux, Mac OS, Windows operating systems. ADMET Predictor requires to purchase a license to be used. An evaluation version of the software can be obtained by full fill a request form available on the ADMET Predictor’s website http://www.simulations-plus.com/software/admetpredictor/.
- ADMET Descriptors allows to assess compounds through queries. ADMET Descriptors is part of the BIOVIA Discovery Studio software. ADMET Descriptors provides to the user through Graphical User Interface (GUI), a set of functions to predict absorption, distribution, metabolism, excretion and toxicity (ADMET) properties starting from a list of molecules. ADMET Descriptors is available for Unix/Linux, Mac OS, and Windows operating systems. To download the trial version, users have to fill the available online form. The download of the complete version of the software requires to purchase a license, and can be done at the following web address http://accelrys.com/products/collaborative-science/biovia-discovery-studio/qsar-admet-and-predictive-toxicology.html.
- PreADMET is a web application for predicting ADME and producing drug-like library using in-silico methods. PreADMET can be freely accessed at the following web address https://preadmet.bmdrc.kr. In the same website, the PreADMET’s purchasing editions are also available. PreADMET consists of four components Molecular-Descriptor-Calculation, Drug-likeness-Prediction, ADME-Prediction, and Toxicity-prediction, and it is developed by using C and PHP programming languages.
- ADMEWORKS ModelBuilder can be used to predict different chemical and biological properties of compounds. ADMEWORKS ModelBuilder provides functions for structural analysis, statistical methods to produce predictive models, outlier detection and analytical functions for features and sample selection. ADMEWORKS ModelBuilder requires to purchase a license to be used; trial version download is possible only filling the registration form. ADMEWORKS ModelBuilder is available at the following web address http://www.fqs.pl/en/chemistry/products/admeworks-modelbuilder.
- ADMEWORKS Predictor is a Web-based application to evaluate the ADMET properties of compounds, available at http://www.fqs.pl/en/chemistry/products/admeworks-predictor web site. ADMEWORKS Predictor provides an interactive graphical 3D structure viewer through a web browser. Also, ADMEWORKS provides the users functions for retrieving, filtering and sorting data.

Table 1 conveys the main features of the summarized software tools. It is worthy to note that, only the first six tools in the Table 1 can extract actionable knowledge hidden in DMET SNP datasets. Regarding other tools, although they refer to DMET, they are not able to analyze DMET SNP datasets but can be employed only to conduct modeling and prediction analysis of the structure of molecules related to DMET.

## 3. Results

To help researchers to perform survival, data mining and statistical analysis in an easy and fast way, we developed and implemented microPipe. microPipe is wrote by using Java 8.0 language. The only requirement to use microPipe on users’ machine is that Java 8 or higher has to be already installed. microPipe has been thought to be easy to use by users with basic or none programming skills through an interactive GUI, as well as to be scalable even when analyzing huge data sets.

microPipe is available under Creative Commons license, and is freely downloadable for academic and not-for-profit institutions at https://sites.google.com/site/micropipeline/.

microPipe is a fast and efficient tool designed to assist the users in statistical analysis, in Kaplan–Meier curve computing/visualization, and in association rules mining from DMET SNP datasets. microPipe comes with an advanced computational engine, which allows to perform data analysis in parallel by taking advantage of the available hardware. microPipe’s engine is designed to exploit the multi-CPUs multi-Cores architectures to speed-up the computational analysis. microPipe automatically maps each phase of the analysis process to a single Core/CPU, and if the available number of available Cores/CPUs. Thus, each analysis step can be done in parallel by exploiting multithread computation, without saturating the system, through some programming methods.

**Figure 1:**
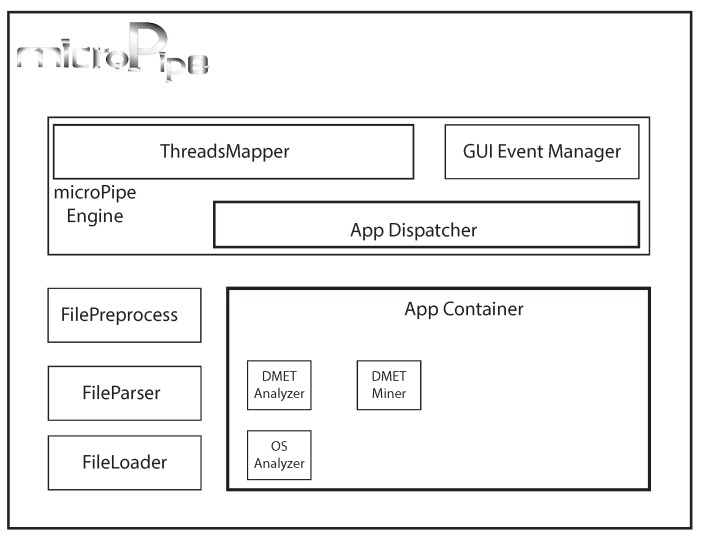
The microPipe’s architecture. GUI: Graphical User Interface; OS: Overall Survival.

The microPipe’s architecture is designed to be efficient as well as simple to be extended by adding new components. The architecture is depicted in Figure 1. Every new component that has been developed implementing the microPipe outlet-interface, can be added by copying it in the microPipe AppContainer. The main modules of microPipe’s architecture are: (i) *FileLoader* loads and checks the consistency of input file; (ii) *FileParser* parses the loaded file, making the data suitable for the next phases; (iii) *FilePreprocess* cleans and deals with data to make data in a format suitable for the application; (iv) *AppDispatcher* receives the preprocessed data and dynamically binds the data with the proper application; (v) *ThreadsMapper* starts the analysis pipeline, where each application can be execute in parallel and independent of the others; (vi) *GUIEventManager* is the graphical control that allow to microPipe and users to interact among them through a simple and intuitive graphical commands.

The microPipe primary function is to avoid that users having to use multiple tools to detect and identify the relevant SNPs involved in different response of subjects to treatment. microPipe can produce heat-map SNPs visualization, designed to quickly highlight the SNPs’ distribution among the subjects involved in the study. Through the Fisher’s Test microPipe can automatically highlight the presence of SNPs related to the clinical conditions of the patient, e.g., the SNPs responsible of the different response to the treatments. Moreover, users by selecting a statistical corrector between Bonferroni and FDR can produce more accurate results, reducing the bias due to the *type* *I* *errors*. microPipe association rule mining is done automatically and in parallel with the other analysis. microPipe demands from the user the setting of parameters for the FisherFilter’s value with which microPipe prunes useless rows, reducing the search space. The setting up of confidence and minimum support values, allows microPipe to show to the user only the relevant association rules that satisfy both values and that are more relevant. Finally, microPipe shows users the probes sorted by statistical relevance obtained computing the log-rank values. In this mode, the user can analyze and save only significant survival curves.

The main feature of microPipe is the capability to perform on the same DMET SNP dataset annotated with temporal events, multiple type of analysis automatically. Moreover, microPipe automatically preprocesses the data through a suitable methodology able to remove useless data, i.e., rows with low information content, and avoiding that users have to repeat manually tedious configuration settings to figure out important clues.

## 4. microPipe Usage Instruction

**Figure 2:**
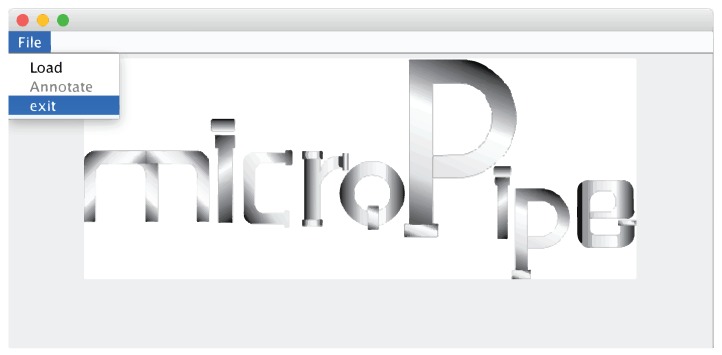
The microPipe’s main window.

In this Section, we describe and use a case involving microPipe. The proposed use case highlights remarkably, the main steps that a user has to follow to get actionable knowledge when analyzing a real DMET SNP dataset. As the first step, the user has to run microPipe. microPipe can be started by double clicking on the file “microPipe.jar,” modality recommended for a user with basic programming skills, or by using the command line. From the command line, expert users can set up specific features of the Java virtual machine, e.g., the heap size, the stack size, to better customize the execution environment. “microPipe.jar” is distributed as Java archive (jar) tool provided as part of the Java Development Kit (JDK) a compressed and executable file. To execute a program distributed as JAR package from the command line, users have to type “*$*java -jar microPipe.jar”. When started microPipe shows the main window (see Figure 2).

microPipe presents an essential and minimal GUI. The GUI comprises only the menu “File” (see Figure 2) containing the Load and exit commands. By clicking on “exit” closes microPipe. Instead, by clicking on “Load” option, it will show the file contained in the user’s hard disk through the chooser window. The file chooser window allow users to browse his/her hard disk to load the input file to study. After locating and selecting the file, by clicking the “ok” button, microPipe automatically starts the analysis. Each step of the analysis pipeline is done in parallel and independently of each other. Survival analysis is done in the background because it does not require input feedback from the user, whereas, since statistical and data mining analysis need some user feedback to produce relevant results, e.g., the values of minimum support to generate association rules are done interactively.

**Figure 3:**
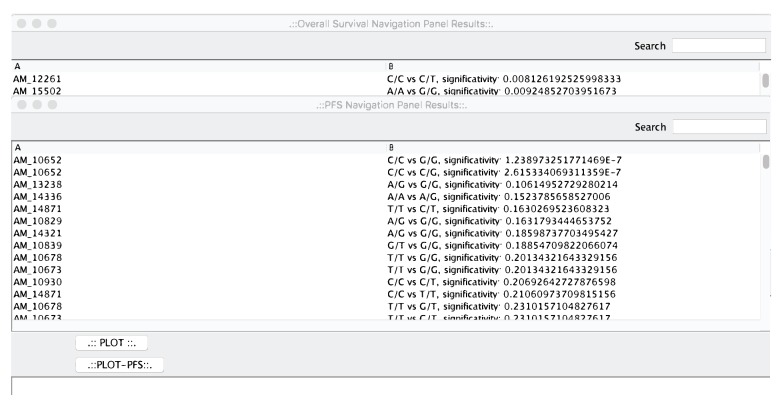
The OS and PFS navigation panels. From that panels, the user can select the most relevant curves to be plotted.

As a first step of the analysis pipeline, microPipe will provide the “OS Navigation Panel Results” and “PFS Navigation Panel Results” windows see (Figure 3), that are the output of survival analysis phase.

**Figure 4:**
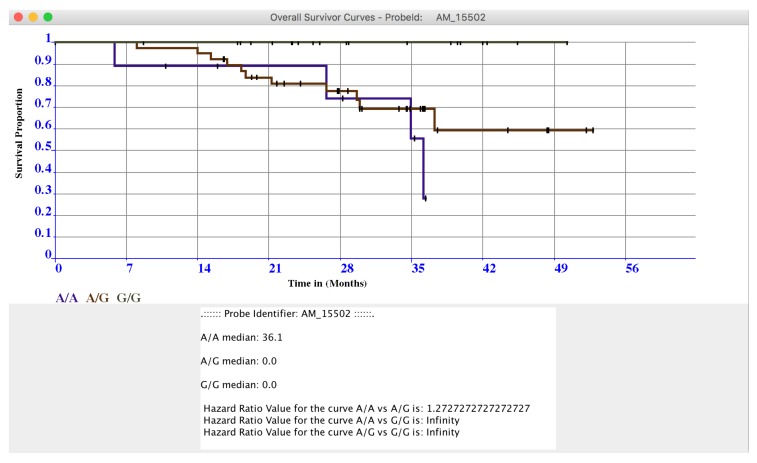
In Figure is presented the OS curve related to the probe AM_15502, obtained by plotting the data in the OS Navigation Panel by clicking on it.

The two windows contain the results sorted by statistical relevance obtained by using the log-rank test. In this way, microPipe provides to the user with a complete view of the most suitable probes that should be further investigated. Conversely from other tools like SPSS, or R, where users have to configure the analysis of the whole dataset manually, by using microPipe all the repetitive configuration steps are done by the software. Thus, users analyze the relevant curves by clicking on each curve and then visualize the related OS or PFS or both curves. If necessary, this chart can be saved on the disk as an image. Moreover, on the bottom of the chart, the medians values and Hazard Ratio Value of each curve are presented, see (Figure 4), highlighting the key features speeding up the understanding of the OS curve.

**Figure 5:**
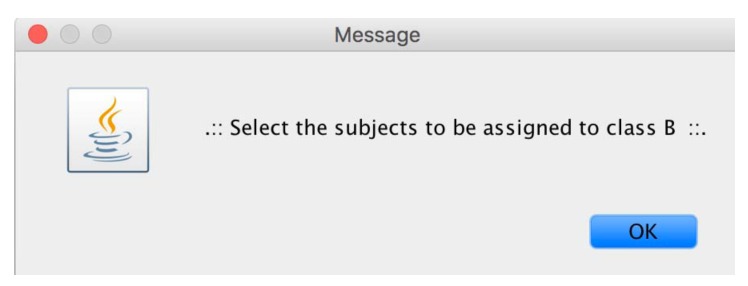
The subjects’ selection message.

Depending on the complexity of the input dataset microPipe warns users to select the subject belonging to class B (see Figure 5). In this way, microPipe signals to the users that the statistical and data mining phases are starting.

**Figure 6:**
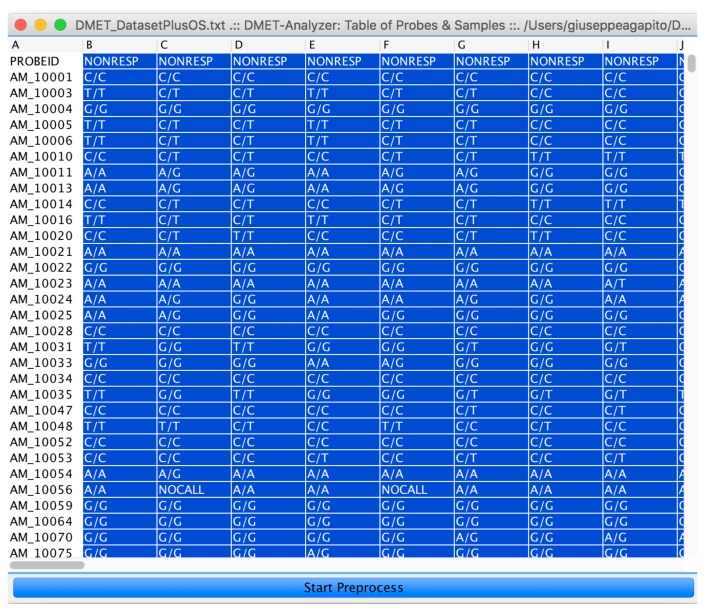
The input DMET SNP dataset represented as an interactive table. By selecting the subject to be assigned to class B and by clicking on the Start Preprocess button, the user can run the microPipe analysis.

After assignment of subjects is done, by clicking on the “preprocess” button (see Figure 6), the user starts statistical and data mining analysis.

**Figure 7:**
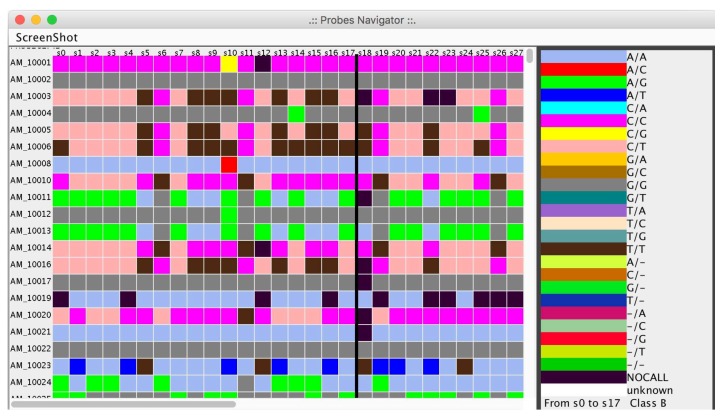
Visualization of the Single Nucleotide Polymorphism (SNP) distribution in each probe represented as a heat map.

As an intermediate step, microPipe will show the heat-map of SNPs’ distribution, and the preprocessed table obtained by filtering useless rows see (Figure 7).

**Figure 8:**
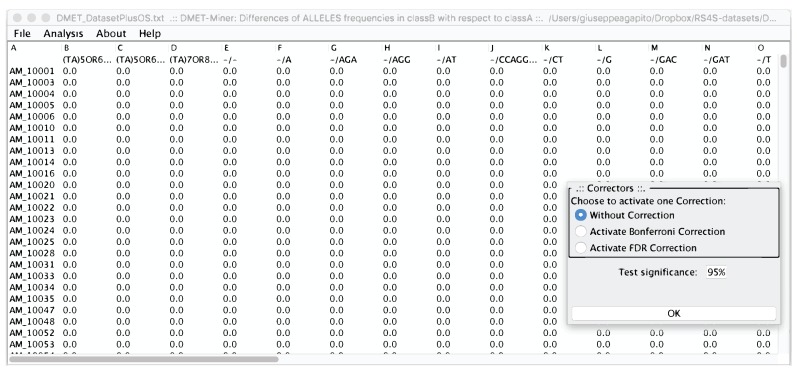
Preprocessed table, from which a user can select the statistical analysis to be performed. In the right corner, the Statistical corrector panel is shown, from which users can decide to apply or not a statistical correction.

From the menu bar in Figure 8, users can perform the Fisher’s Test on all the pre-processed table, or chose to compute the Fisher’s Test on a specific probe or to calculate the Hardy-Weinberg equilibrium.

**Figure 9:**
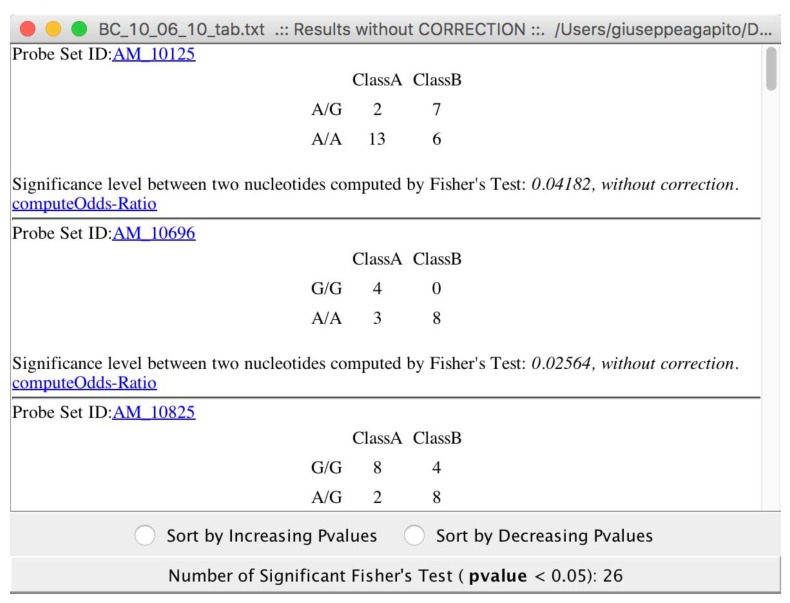
The relevant SNPs detected from microPipe by using Fisher’s Test.

Choosing to compute “exaustiveFisherTest” with or without correctors, all the relevant Fisher test are conveyed see (Figure 9).

**Figure 10:**
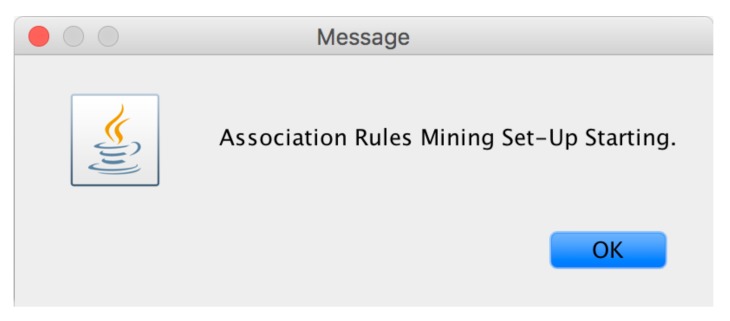
Association Rule Mining warning message. microPipe advices the users that the mining phase is starting up.

The results of Fisher tests allow users to discover SNPs related to the particular disease, or treatment under investigation (see Figure 9). As the final step of the analysis pipeline, microPipe will inform the user that it is setting up association rules and mining, see Figure 10.

**Figure 11:**
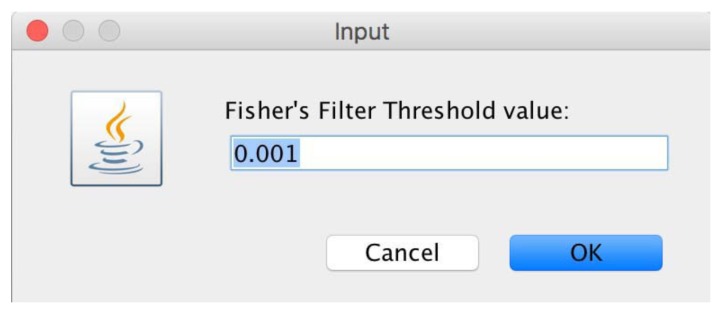
Window from which users can define the FisherFilter value with which to remove all the useless rows below this value.

microPipe will ask users to choose the FisherFilter value that is different from that used in the statistical analysis (see Figure 11) with which to remove as many as rows possible that are under the chosen threshold value.

**Figure 12:**
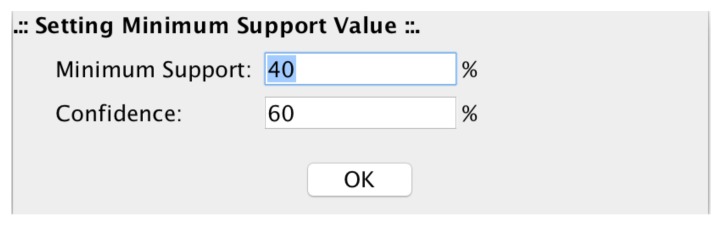
Minimum Support and Confidence setting up panel. In this panel, users can set the values of Minimum Support and Confidence with which microPipe computes the strong assocition rules.

The filtering value is automatically computed for every single row (probe). The setting up of confidence and minimum support values, allows microPipe to show to the user only the strong association rules that at least meet both values (see Figure 12).

**Figure 13:**
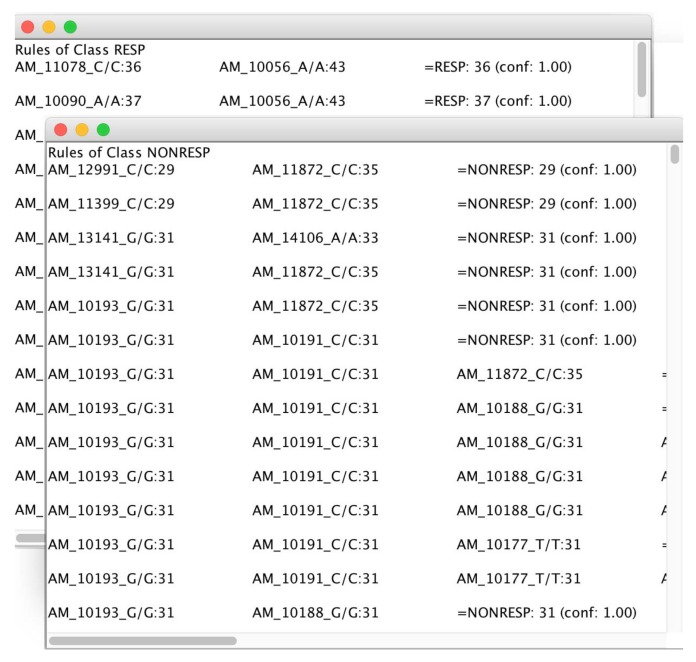
Association rules mined by microPipe and conveyed in two windows related to the two analyzed classes i.e., Responding and Not-Responding in short RESP and NoRESP.

As the final step, the mined rules are split and presented in two separate windows and ranked by confidence value, as conveyed in Figure 13.

The rules mined by microPipe, allow users to figure out the multiple relations among SNPs that are responsible for a particular response to a drug for one class in a pharmacogenomics study.

In summary, microPipe is a useful tool to conduct multiple analysis through a few mouse clicks.

It make it possible to analyze an entire DMET SNP dataset with statistical and data mining techniques only by uploading the file to be examined.

Indeed, this methodology frees researchers from having to spend time in repetitive steps such as adapting the dataset, preprocessing data, and so on that are tedious and error-prone tasks. Alternatively, by using microPipe, researchers can devote themselves exclusively to the analysis of results and their interpretation, in a much shorter time. Table 2 reports the execution times and the used memory needed to accomplish a DMET SNP analysis workflow by using each tool alone, or by using the novel microPipe system. Analyzing the data reported in Table 2, it is worth to note that, by using microPipe in a single run, it is possible to accomplish the whole analysis workflow in half the time or less than using the three software individually. On the other hand, the memory used by microPipe is slightly greater than the sum of the memory used by the three tools. The data reported in Table 2 refer to the time necessary to locate the dataset on the user hard disk up to the visualization of the final results. For all the tools, we used the same settings to detect the execution times and memory usage, i.e., we used the same dataset, the same value of minimum support and confidence, and used the same location on the hard disk from which to load the input dataset.

The effectiveness of microPipe in supporting personalized medicine is mainly related to its ability, in pharmacogenomics studies, to find correlations between the genotype of individuals (e.g., the presence of SNPs in ADME genes found by the DMET microarray), and the response to drugs (e.g., toxic or non toxic effect). After clinical validation, this would allow a better choice of drugs on the basis of individual genotype. Moreover, its ability to correlate SNPs to overall survival is another advantage of microPipe. The strength of the microPipe methods to produce new knowledge useful in personalized medicine, has been proven in different previous works in the oncology field [4,5,6].

## 5. Conclusions

This paper presented microPipe, a software pipeline that allows users to perform different analyses on DMET data, such as data mining, statistical and survival analysis through some mouse clicks. The easiness of microPipe makes it possible to speed up the investigation of DMET SNP data, because users are not involved in tedious and error prone tasks. The main strength of microPipe is the advanced computational engine that allows to perform several data analyses in parallel, by taking advantage of the available hardware. microPipe’s engine is designed to exploit the multi-CPUs multi-Cores architectures to speed-up the computational analysis. The primary goal of microPipe is to support researchers in their daily activities, allowing them to analyze substantial heterogeneous datasets through several different kinds of analysis efficiently, without the need to manually check if the data are compatible with the chosen tools. The manual manipulation of a dataset is a tedious and error-prone, and could introduce bias into the data and consequently could produce poor accurate results. Conversely, by using microPipe, all the repetitive phases are done automatically by the tool. Thus, researchers can focus only on the analysis and evaluation of results, speeding up the process of knowledge extraction.

## Figures and Tables

**Table 1:** Comparison of the functionality provided by the tools compatible with Drug Metabolism Enzymes and Transporters (DMET) reviewed on OMICtools website. WUML is short for Windows, Unix, Mac, and Linux. na means that the information was not available. WebApp refers to a web application that can be used through a web browser. The *√* symbol indicates that the tool provides that function, whereas × means an unsupported feature.

TOOL	Operating System	Statistical	Data Mining	Prediction	Modelling	Free	Pay
DMET-Analyzer	WUML	*√*	×	×	×	*√*	×
DMET-Miner	WUML	×	*√*	×	×	*√*	×
OS-Analyzer	WUML	*√*	×	×	×	*√*	×
coreSNP	WUML	*√*	×	×	×	*√*	×
PARES	WUML	×	*√*	×	×	*√*	×
cloud4SNP	WUML	*√*	×	×	×	*√*	×
ADMET Predictor	WUML	×	×	*√*	×	×	*√*
ADMET Descriptors	WUML	×	×	*√*	×	×	*√*
PreADMET	WebApp	×	×	*√*	×	×	*√*
ADMEWORKSModelB	na	×	×	*√*	*√*	×	*√*
ADMEWORKSPredictor	WebApp	×	×	*√*	×	×	*√*

**Table 2:** microPipe, DMET-Analizer, DMET-Miner, and OS-Analyzer execution times and used memory.

Tool	Time to Complete Analysis (s)	Used Memory (MB)
DMET-Analizer	50	22.8
DMET-Miner	60	24.5
OS-Analyzer	20	18.7
microPipe	52	66.5

## Abbreviations

The following abbreviations are used in this manuscript:

SNP	Single Nucleotide Polymorphism
DMET	Drug Metabolism Enzymes and Transporters
ADME	Adsorption, Distribution, Metabolism and Excretion
NGS	Next Generation Sequencing
GWAS	Genome-Wide Association Studies
MS	Mass Spectrometry
OS	Overall Survival
PFS	Progression Free Survival
JDK	Java Development Kit
jar	Java Archive
FDR	False Discovery Rate
GUI	Graphical User Interface
ARFF	Attribute-Relation File Format
CSV	Comma Separated Values
OSA	OS-Analyzer
FP-Growth	Frequent Pattern Growth

References
